# Intergranular Corrosion and Microstructural Evolution in a Newly Designed Al-6Mg Alloy

**DOI:** 10.3390/ma14123314

**Published:** 2021-06-15

**Authors:** Kweon-Hoon Choi, Bong-Hwan Kim, Da-Bin Lee, Seung-Yoon Yang, Nam-Seok Kim, Seong-Ho Ha, Young-Ok Yoon, Hyun-Kyu Lim, Shae-Kwang Kim

**Affiliations:** 1 Department of Industrial Materials and Smart Manufacturing Engineering, Korea University of Science and Technology, Daejeon 34113, Korea; kchoi74@kitech.re.kr; 2 Advanced Process and Materials R&BD Group, Korea Institute of Industrial Technology (KITECH), Cheonan 31056, Korea; dabin25@kitech.re.kr (D.-B.L.); sy8357@kitech.re.kr (S.-Y.Y.); kimns@kitech.re.kr (N.-S.K.); shha@kitech.re.kr (S.-H.H.); veryoon@kitech.re.kr (Y.-O.Y.); hklim@kitech.re.kr (H.-K.L.); shae@kitech.re.kr (S.-K.K.)

**Keywords:** light alloys, Al-Mg, high-strength, mechanical properties, intergranular corrosion, precipitation

## Abstract

In this work, the microstructure and corrosion behavior of a novel Al-6Mg alloy were investigated. The alloy was prepared by casting from pure Al and Mg+Al_2_Ca master alloy. The ingots were homogenized at 420 °C for 8 h, hot-extruded and cold-rolled with 20% reduction (CR20 alloy) and 50% reduction (CR50 alloy). The CR50 alloy exhibited a higher value of intergranular misorientation due to a higher cold rolling reduction ratio. The average grain sizes were 19 ± 7 μm and 17 ± 9 μm for the CR20 and CR50 alloys, respectively. An intergranular corrosion (IGC) behavior was investigated after sensitization by a nitric acid mass-loss test (ASTM G67). The mass losses of both the CR20 and CR50 alloys were similar at early periods of sensitization, however, the CR20 alloy became more susceptible to IGC as the sensitization time increased. Grain size and β phase precipitation were two critical factors influencing the IGC behavior of this alloy system.

## 1. Introduction

The 5xxx series Al-Mg alloys are widely used materials in the automotive industry due to their high strength-to-weight ratios, weldability, and good corrosion resistance [1,2,3]. Strength and ductility can be improved by adding solute Mg to the alloys because of the solid solution strengthening mechanism [4,5]. Nevertheless, as the amount of Mg increases, a selective oxidation prevails and causes a formation of oxide inclusions at elevated temperatures [6]. The rapid oxidation of magnesium alloys can be suppressed by additions of Ca [7]. With this background, a new alloying strategy has been developed. The strategy uses an Mg + Al_2_Ca master alloy instead of pure Mg during casting. The Mg + Al_2_Ca master alloy improves the oxidation resistance of Al-Mg alloys by forming a protective CaO/MgO mixed layer on the surface [8,9]

Previous studies have shown that anodic β-phase (β-Mg_2_Al_3_) precipitation along grain boundaries is an important factor affecting the intergranular corrosion (IGC) susceptibility of Al-Mg alloy [6,7,8]. The Mg segregation leads to anodic β-phase formation at 50–200 °C. The β-phase precipitation is referred to as sensitization [10,11,12,13]. The β-phase formation is usually observed at grain boundaries (GB), sub-grains, intermetallics, dislocations and other defects. Generally, the sequences of β precipitation have been reported as follows [14,15,16]:

Solid solution α → Guinier–Peston zones → β’’ → β’ → β precipitation

The β phases are electrochemically active compared to the Al matrix which leads to galvanic corrosion. The distribution and morphology of β precipitates affect the susceptibility of the alloy to IGC. To evaluate the IGC susceptibility, a nitric acid mass-loss test (NAMLT, ASTM G67) can be used to measure the corrosion rate.

In this research, the IGC susceptibility of sensitized Al-6Mg alloy with two different cold rolling conditions was studied. It is not yet clearly understood how the cold rolling process affects the corrosion behavior [17]. D’Antuono et al. observed that an increased rolling reduction increased the growth rate of β precipitation due to lowering nucleation temperature [18]. Additionally, an initial β precipitation was observed preferentially at low-angle grain boundaries rather than high-angle grain boundaries [19]. On the contrary, Wang et al. found that the maximum corrosion depth decreases with increasing cold rolling reduction ratio. They also stated that larger thickness reductions are attributable to an increased number of small-sized grains formed at the grain boundary, which can eventually break off the continuity of corrosion.

This paper aims to figure out the effect of cold rolling and sensitization treatment on the IGC susceptibility of a newly designed Al-6Mg alloy. In this study, a microstructure evolution of the alloy was analyzed by electron backscattered diffraction (EBSD). The continuity of β-phase precipitation at grain boundary was studied by scanning electron microscopy (SEM) and transmission electron microscopy (TEM). The effects of the cold rolling reduction ratio and sensitization heat treatment on the IGC susceptibility are discussed.

## 2. Materials and Methods

### 2.1. Sample Preparation

The ingots of Al-6Mg alloy were prepared in an electric resistance furnace by the casting of pure Al and Mg + Al_2_Ca master alloy. The ingots were homogenized at 420 °C into a plate with an extrusion ratio of 21.7:1. The initial extrusion thickness was 12 mm. The alloy was cold-rolled to 3 mm by using a 2-high mill rolling machine (FENN, East Berlin, CT, USA). The cold-rolled specimen was further annealed at 420 °C for 1 h in a box furnace and air-cooled. Finally, the alloy was cold-rolled with 20% reduction (CR20 alloy) and 50% reduction (CR50 alloy), respectively. The chemical composition of the Al-6Mg alloy is shown in Table 1. The chemical composition was measured by inductively coupled plasma-atomic emission spectroscopy (ICP-AES, SPECTRO).

### 2.2. Intergranular Corrosion Test (NAMLT)

The specimens were sensitized at 100 °C for 0, 3, 7, 48, 144, and 207 h prior to corrosion testing. A nitric acid mass-loss test (NAMLT, ASTM G67) was used to determine the IGC susceptibility [20]. According to the standard, the test specimen was machined into blocks. The surfaces of the blocks were polished with 320 grit abrasive paper, and the specimen dimensions were measured to the nearest 0.02 mm. Before the test, the samples were etched in 5% NaOH (1 min at 80 °C). The initial mass of each specimen was measured on a digital scale. The samples were then immersed in 70% HNO_3_ for 24 h. After the test, all specimens were carefully rinsed with water and brush with a stiff plastic brush. The final weight of the specimens was measured and used to calculate the mass-loss rate [20].

Microstructural evolutions of the alloys were investigated by optical microscopy (OM, Nikon MA200, Nikon, Tokyo, Japan), scanning electron microscopy (FE-SEM/EDSFEI Quanta200F, Hitachi, Japan), electron backscatter diffraction (EBSD, EDAX Hikari EBSD detector, Mahwah, NJ, USA), and transmission electron microscopy (TEM, JEOL JEM-2100F, Akishima, Japan). The obtained EBSD data were analyzed using the MTEX software (open-source MATLAB toolbox) [21]. Image J software (open-source Java image program, NIH Image) was used to calculate the maximum intergranular corrosion depth and area.

## 3. Results

### 3.1. Microstructure

**Figure 1:**
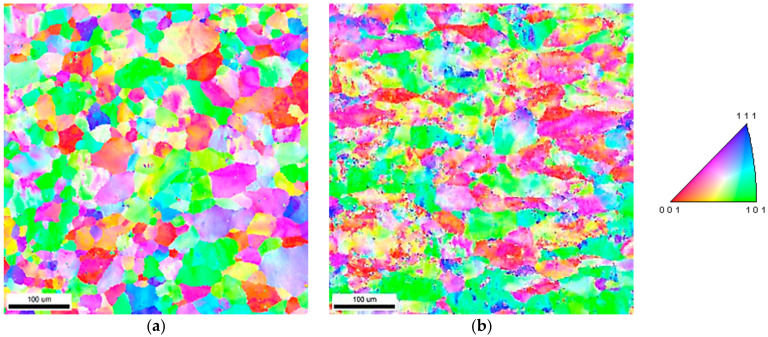
Inverse pole figure (IPF) maps of the new Al-6Mg CR20 (**a**) and CR50 alloys (**b**).

**Figure 2:**
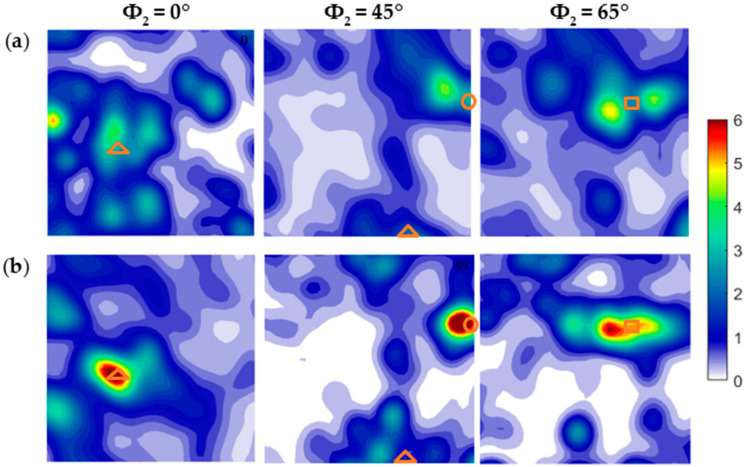
ODF sections of the new Al-6Mg CR20 (**a**) and CR50 alloys (**b**). The triangle represents Brass texture, circle is Copper texture, and the square is used for S texture.

Inverse pole figure (IPF) maps of newly developed Al-6Mg with two different cold rolling conditions are shown in Figure 1. The results reveal that the CR20 alloy has more equiaxed grains compared to the CR50 alloy. The CR50 alloy has a deformed microstructure due to a higher cold rolling reduction ratio. Figure 2 shows the orientation distribution functions (ODF) of the CR20 and the CR50 alloy. Typical deformation textures of the cold-rolled Al-6Mg alloys are also shown in Figure 2. As the cold rolling reduction ratio increased, more deformed textures tended to be obtained.

Miller indices of the texture components for rolled samples are listed in Table 2. In the previous studies it was reported that the fraction of deformation texture components (Brass {110} <112>, Copper {112} <111> and S {123} <634>) increased while the recrystallization texture (Cube {100} <010> and Goss {011} <100>) did not change with increasing cold rolling reduction ratio in the Al-Mg alloy [22,23]. Figure 2a indicates that the CR20 alloy has an evolution of copper texture in Φ2 = 45 section. The CR50 alloy shows a strong copper texture (Figure 2b). In addition, the CR50 alloy shows a stronger brass and S texture compared to the CR20 alloy. The results are in accordance with previous research [22,23]. However, the recrystallization texture was not found in the newly designed Al-6Mg alloy, which shows a partial disagreement with previous studies [22,23]. Therefore, the texture of the newly developed Al-6Mg alloy is yet to be completely understood.

**Figure 3:**
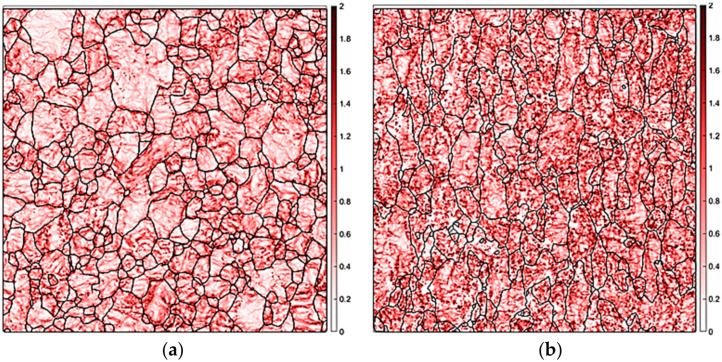
KAM maps of the CR20 (**a**) and CR50 alloys (**b**).

**Figure 4:**
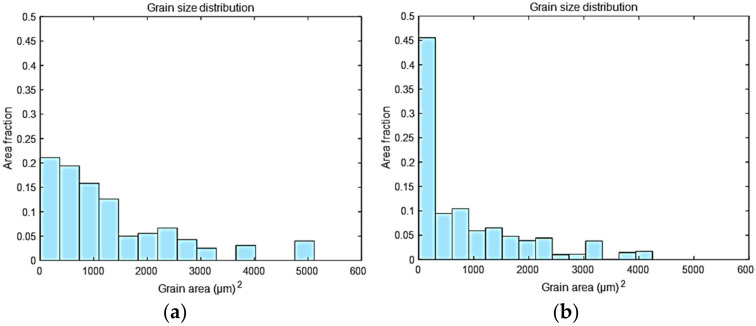
Grain size area histograms of the CR20 (**a**) and CR50 alloys (**b**).

Kernel average misorientation (KAM) is a measure of local grain misorientation based on the set of all neighboring misorientation. This map can be used to explain the effect of the rolling reduction ratio on the intergranular misorientation and evaluate the stored strain energy for a given point [24]. Figure 3 shows that the KAM map of the CR20 and CR50 alloys. The CR50 alloy exhibits a higher value of intergranular misorientation. As the reduction ratio of cold rolling increases, the dislocation density and volume fraction of low-angle grain boundary (LAGB) increase, which results in increasing KAM value. Figure 4 illustrates the grain size areas of CR20 and CR50 alloys. The average grain size areas of the CR20 and CR50 alloys were calculated as 365.8 ± 51.21 μm^2^ and 283.6 ± 87.92 μm^2^, respectively. These values correspond to average grain sizes of 19 ± 7 μm for the CR20 alloy and 17 ± 9 μm for the CR50 alloy, respectively.

### 3.2. Mass-Loss Test Results

**Figure 5:**
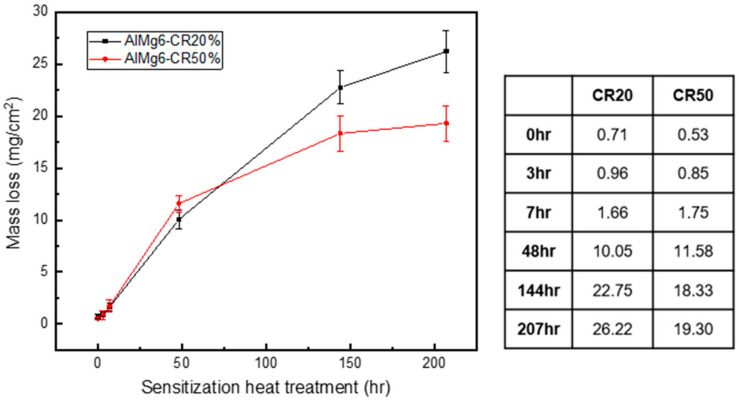
NAMLT results of the CR20 alloy and CR50 alloy with different aging times at 100 °C.

Figure 5 shows the NAMLT results of the CR20 and CR50 alloys. The specimens were sensitized at 100 °C for 0, 3, 7, 48, 144, and 207 h prior to corrosion testing. The figure shows that both the CR20 and CR50 alloys had similar mass losses in concentrated nitric acid at sensitization for less than 7 h. As the sensitization time increased, the CR50 showed a slightly smaller mass-loss rate compared to the CR20 alloy. A previous study indicates that the IGC susceptibility of the Al alloys gradually decreases as the cold rolling reduction ratio increases [25].

The mass-loss of 25 to 75 mg/cm^2^ indicates that the specimen is susceptible to IGC. Smaller values correspond to better corrosion resistance. The effect of the manufacturing process on the IGC of Al-Mg alloys has been previously studied [25,26,27]. Zhang et al. studied how the grain size modification by various manufacturing processes affected the intergranular corrosion [26]. Previous researchers reported that the NAMLT value of AA5083 alloy (~4.5 wt% of solute Mg) was 18 mg/cm^2^ after 8 days (192 h) of sensitization exposure at 100 °C [27]. Similar values (19 mg/cm^2^ after 200 h of sensitization at 100 °C) were obtained in the current study. Therefore, the IGC susceptibility of high Mg-containing Al-Mg alloys appears not to be negatively affected by the cold rolling process.

### 3.3. Microstructure of Corrosion Attacked Surface

**Figure 6:**
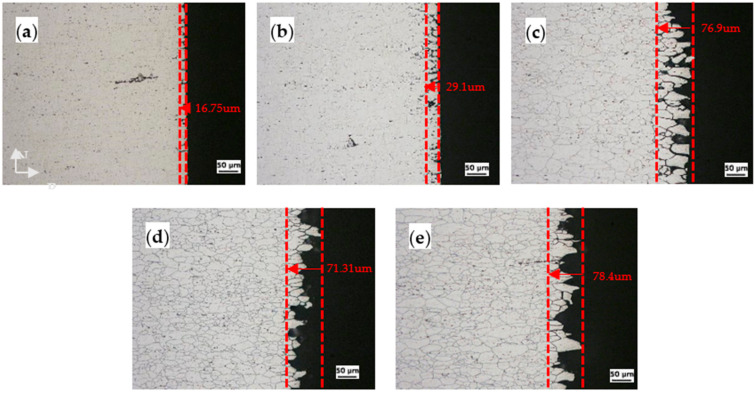
The surface of the CR20 alloy after NAMLT at different sensitization times: (**a**) 0 h, (**b**) 7 h, (**c**) 48 h, (**d**) 144 h, and (**e**) 207 h.

**Figure 7:**
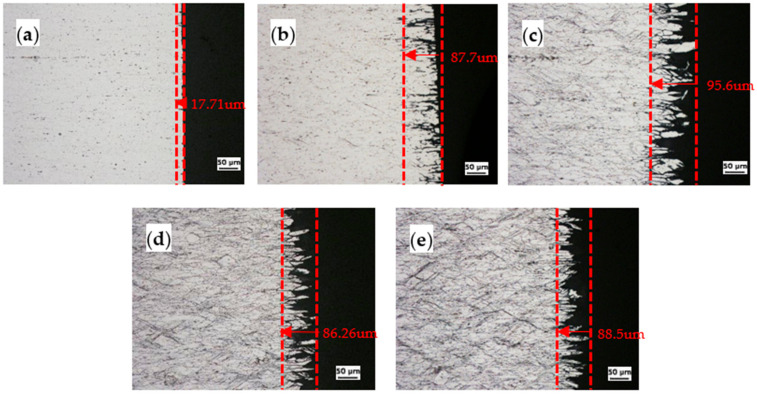
The surface of the CR50 alloy after NAMLT at different sensitization times: (**a**) 0 h, (**b**) 7 h, (**c**) 48 h, (**d**) 144 h, and (**e**) 207 h.

Figure 6 and Figure 7 show the metallographic cross sections of the CR20 and CR50 alloys after the IGC test. To explore the IGC behavior, the specimens were etched using Keller’s reagent for 10 s. Figure 6a and Figure 7a indicate the estimated corrosion depths after 0 h of sensitization. The depths are 16.75 μm and 17.71 μm for the CR20 and CR50 alloys, respectively. The penetration depth increases with increasing annealing time. The precipitation of β at grain boundaries increases with increasing sensitization time. It is assumed that the anodic precipitation of the CR50 alloy is deeper than in the CR20 alloy, which results in the IGC rate being higher in this alloy. As shown in Figure 6c and Figure 7c, the grains start to be detached from the specimen after 48 h at 100 °C.

**Figure 8:**
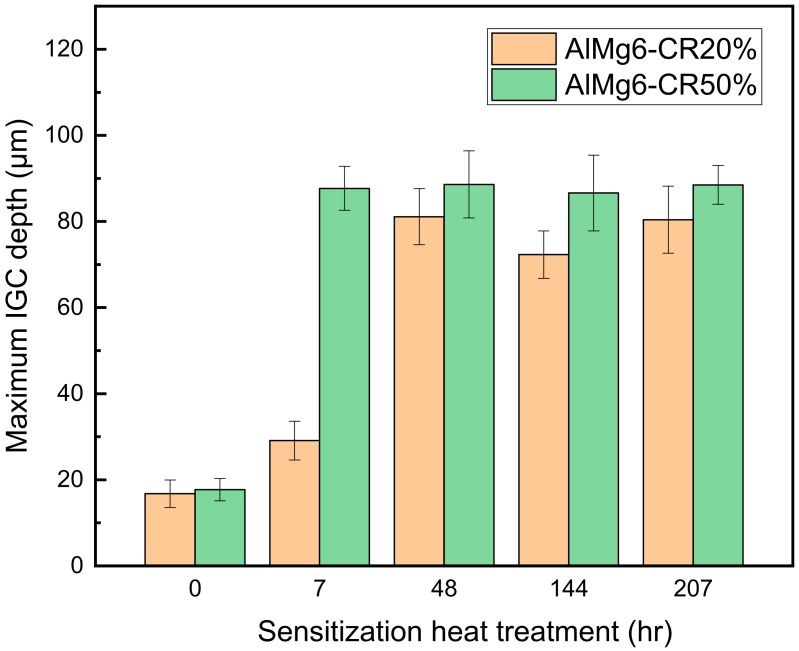
Maximum IGC depths of CR20 and CR50 alloys.

Figure 8 shows the maximum corrosion depth of both CR20 and CR50 alloys. The values are similar at the maximum sensitization time. On the other hand, the CR20 alloy shows a higher mass-loss rate compared to the CR50 alloy (Figure 5).

The continuity of β precipitation at grain boundary is critical for the IGC depth. The grain size, on the other hand, is a more important factor in the mass-loss rate at the early periods of sensitization. While there is not a significant difference between the alloys in maximum IGC depth, the NAMLT results show a higher mass-loss rate in the CR20 alloy after the long heat treatment time. This means that the CR20 alloy is more susceptible to IGC.

### 3.4. Schematic of β Phase Distribution in Grain Boundary

**Figure 9:**
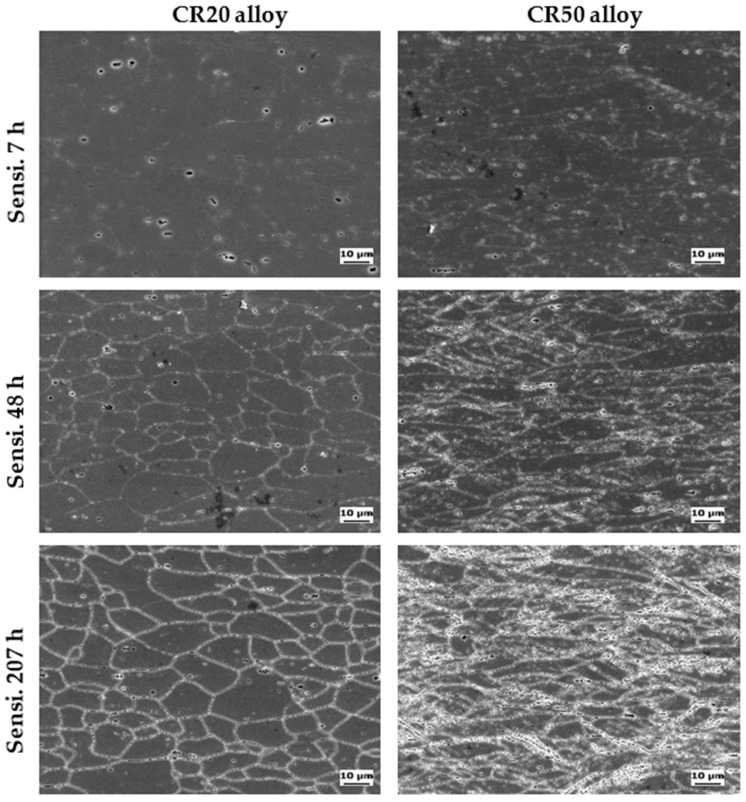
Microstructure of sensitized CR20 and CR50 alloys.

**Figure 10:**
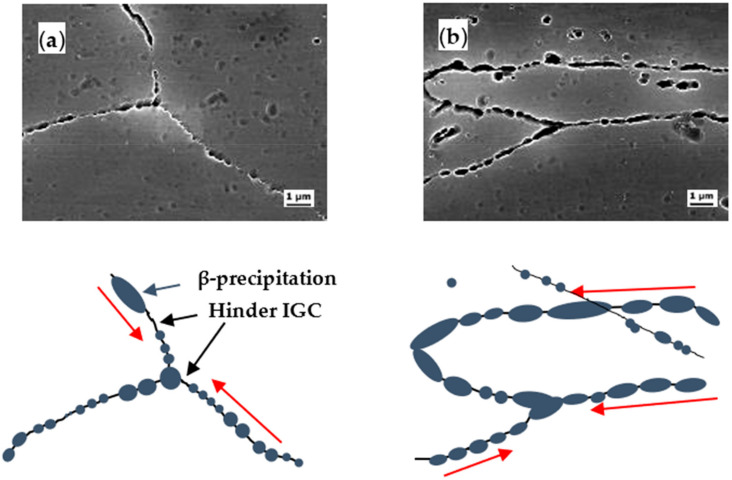
Close inspection of β precipitation covered grain boundary: (**a**) CR20 alloy, Sensi. 48 h, (**b**) CR50 alloy, Sensi. 48 h.

**Figure 11:**
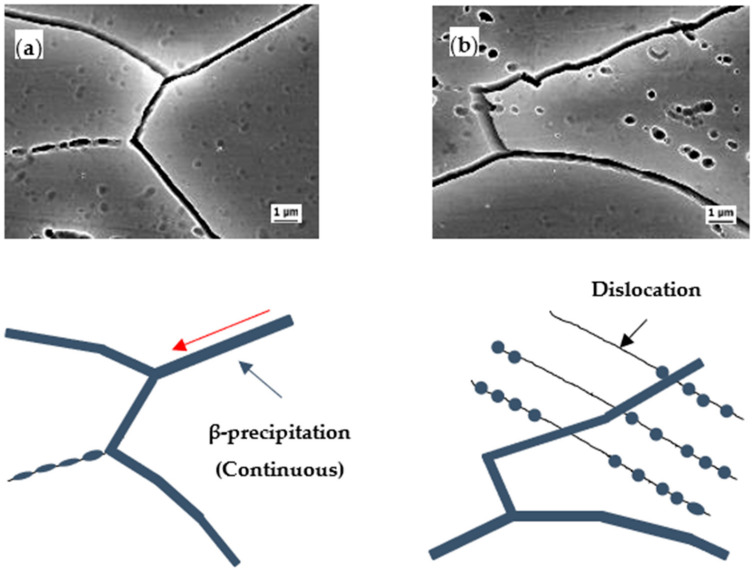
Close inspection of β precipitation covered grain boundary: (**a**) CR20 alloy, Sensi. 207 h, (**b**) CR50 alloy, Sensi. 207 h.

Figure 9 shows the SEM images of the CR20 and CR50 alloy surface after sensitization. The grain boundary is covered with β precipitation. The specimens were etched using H_3_PO_4_ etchant to selectively reveal the grain boundary covered by the anodic β precipitation. The continuity of β precipitation can be indirectly observed by the microstructure of the etched surface. At sensitization time of 7 h, the grain boundaries were more discernible in the CR50 alloy. This indicates that the CR50 alloy is more susceptible to IGC at 7 h sensitization. Figure 10 and Figure 11 show a close look-up at the microstructure of the etched surface. The grain boundary is covered by β precipitation. The β precipitation is more discontinuous in the CR20 alloy.

Previous studies found that the precipitate growth rates increased with rolling reduction [18,19]. A high density of dislocations can lower the activation energy, which most likely initiates the precipitation in the rolled specimen [18]. Increasing the dislocation density results in enhancing the diffusivity of Mg atoms at sensitization treatment due to pipeline diffusion [19]. On the other hand, there is no difference in the continuity of the precipitation at grain boundary at long sensitization times (Figure 11) by precipitation [18]. Considering both mass-loss results and maximum IGC depths (Figure 5 and Figure 9), the grain size thus becomes a crucial factor in IGC at longer sensitization times.

### 3.5. Direct Observation of β Precipitation Distribution

**Figure 12:**
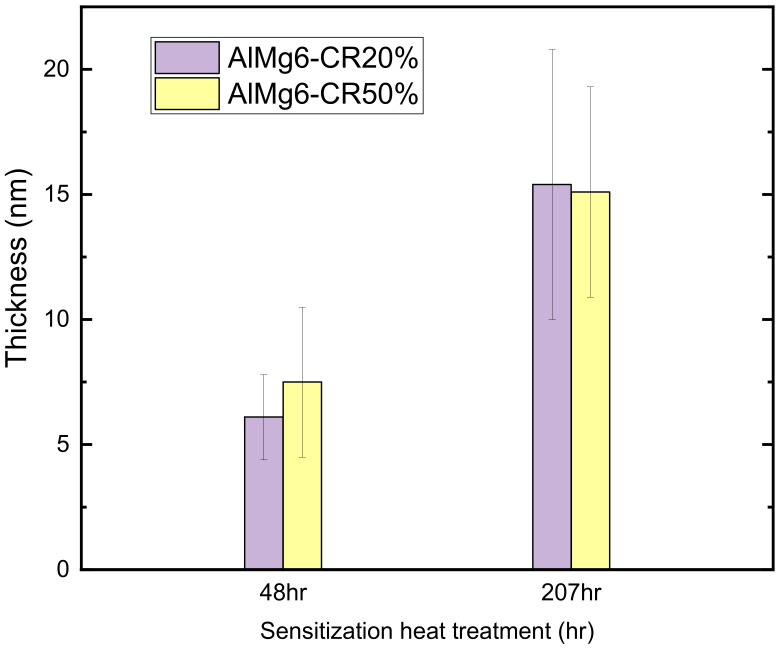
Thickness of β-phase precipitates in the CR20, and CR50 alloys sensitized for 48 and 207 h, respectively.

**Figure 13:**
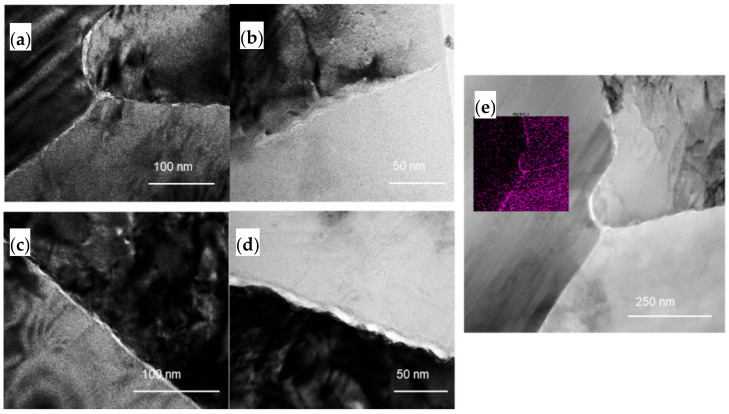
TEM of β precipitates in: (**a**) CR20 alloy, 48 h; (**b**) CR50 alloy, 48 h; (**c**) CR20 alloy, 207 h; (**d**) CR50 alloy, 207 h; and (**e**) TEM-EDS (Mg element) CR20 alloy, 48 h.

Figure 12 shows the thickness of the precipitates for the CR20 and CR50 alloys. The size of the precipitates was calculated from the crossline thickness of the precipitates by TEM image (Figure 13). Figure 13e represents the TEM image of the CR20 alloy with EDS mapping of magnesium at 48 h of sensitization. The thickness was found to be 6.1 ± 1.7 nm for the CR20 alloy and 7.5 ± 3.0 nm for the CR50 alloy at 48 h of sensitization time. The precipitate thickness at 207 h is higher. The thickness of β-precipitates is crucial for the IGC rate as it affects the continuity of the precipitation at the grain boundary. Figure 13a reveals that the β precipitation of the CR20 alloy in the early period of sensitization is discontinuous. This results in a superior corrosion resistance compared to the CR50 alloy at the early sensitization time. The size of β precipitates at longer sensitization times becomes larger for the CR20 alloy. Both Figure 13c,d show that β precipitation was almost continuously distributed at the grain boundary with almost the same thickness as shown in Figure 12. Zhang et al. also agree that the kinetics of precipitation growth is reduced with sensitization time [28]. The results of this study show that the IGC significantly depended on the grain size for long-term sensitization, as compared to the size of precipitates.

## 4. Discussion

In this research, we found that both grain size and continuity of β precipitation at grain boundaries are important factors affecting the Al-Mg IGC susceptibility.

The TEM image in Figure 13 shows that the β precipitates are much thicker in the CR50 alloy at an early period of sensitization. The β precipitates thickness, however, is almost the same at long-term sensitization. Some researchers suggested that grain boundary misorientation is a crucial factor for the growth rate and the final size of β precipitation. These factors affect the continuity of β precipitation at grain boundaries [10,12,26,29,30,31,32,33,34]. Wang et al. concluded that some grain boundaries, e.g., low-angle grain boundaries generated by plastic deformation, are not susceptible to IGC [28]. On the other hand, D’Antuono reported that although the β precipitation was preferentially formed at low-angle grain boundary, the final size of precipitation was larger at high-angle grain boundary [18]. The influence of grain boundary plane orientation was reported to affect the continuity of precipitation. It was found that grain boundary (GB) planes close to {110} direction facilitate the β precipitation while the GB plane near {100} direction may be resistant to β precipitation [31,32].

Previous studies showed that the rolled specimen had a high resistance to IGC coming from the confluence of refined grain size and the fraction of low-angle grain boundaries [17,25,26]. In this study, it was revealed that the effect of grain size on IGC needs to be considered depending on the sensitization heat treatment which affects the formation of anodic β-Mg_2_Al_3_ precipitation at the grain boundary. High dislocation density induced by cold rolling facilitates the precipitate growth rates. The formation of anodic β-Mg_2_Al_3_ is affected by temperature and the presence of prior strain [35,36]. The increased dislocation density tends to lower the nucleation temperature and reduce Mg diffusion at a lower temperature [18,19]. These factors are reflected in increasing the susceptibility of the CR50 alloy in the early period of sensitization. In this situation, the dislocation density and grain boundary type affect the IGC susceptibility more significantly compared to the grain size. On the other hand, the grain size affects the IGC susceptibility of cold-rolled Al-6Mg alloy more dramatically than the grain boundary type. It was found that the large-grained material tends to be more susceptible to IGC when the precipitation is continuously formed at the grain boundary due to sufficient sensitization time.

## 5. Conclusions

This study has explored the IGC behavior of a newly designed Al-6Mg alloy with two different cold rolling conditions. It was revealed that the grain size and the continuity of β precipitation play an important role in IGC. The precipitation growth rate and final size of precipitates affect how the grain boundary is covered by β precipitation. At the early period of sensitization, the precipitation growth rate is a crucial factor in IGC. The dislocation density and grain boundary orientation affect the precipitation growth rate. The CR50 alloy has a slightly higher precipitation growth compared to the CR20 alloy because of the high density of dislocations. This results in a higher maximum IGC depth. However, the grain size effect is more dominant when the sensitization time is long enough to cover the grain boundary by anodic β precipitation.

## Figures and Tables

**Table 1:** Chemical composition of Al-6Mg alloy (wt.%, measured by ICP-AES).

Alloy	Si	Fe	Cu	Mg	Mn	Ca	Al
Al-6Mg	0.05	0.07	<0.01	6.04	0.03	~0.02	Bal.

**Table 2:** List of texture components for cold-rolled samples.

Type	Components	Orientation
Recrystallization textures	Cube	{100} <010>
	Goss	{011} <100>
Deformation textures	Brass	{011} <211>
	S	{123} <634>
	Copper	{112} <111>

## Data Availability

Data sharing not applicable. No new data were created or analyzed in this study. Data sharing is not applicable to this article.
